# Longitudinal patterns of intermittent oral corticosteroid therapy for asthma in the United Kingdom

**DOI:** 10.1016/j.jacig.2024.100225

**Published:** 2024-02-02

**Authors:** Trung N. Tran, Heath Heatley, Jennifer Rowell, Jeffrey Shi Kai Chan, Arnaud Bourdin, Jatin Chapaneri, Benjamin Emmanuel, Danny Gibson, David J. Jackson, Andrew N. Menzies-Gow, Ruth Murray, Derek Skinner, David B. Price

**Affiliations:** a BioPharmaceuticals Medical, AstraZeneca, Gaithersburg, Md; b Observational and Pragmatic Research Institute, Singapore; c AstraZeneca, Cambridge, United Kingdom; d Department of Respiratory Diseases, PhyMedExp, University of Montpellier, Montpellier, France; e Guy’s and St Thomas’ NHS Trust and School of Immunology & Microbial Sciences, King’s College, London, United Kingdom; f UK Severe Asthma Network and National Registry, Royal Brompton & Harefield Hospitals and School of Immunology & Microbial Sciences, King’s College, London, United Kingdom; g Respiratory and Immunology, BioPharmaceuticals Medical, AstraZeneca, Cambridge, United Kingdom; h Optimum Patient Care, Cambridge, United Kingdom; i Centre of Academic Primary Care, Division of Applied Health Sciences, University of Aberdeen, Aberdeen, United Kingdom

**Keywords:** OCS, intermittent, asthma, risk, prescription

## Abstract

**Background:**

Increasing frequency of intermittent oral corticosteroid (OCS) prescription and cumulative OCS exposure increase the risk of OCS-related adverse outcomes.

**Objective:**

We sought to describe the evolution and trajectory of intermittent OCS prescription patterns in patients with asthma and investigate risk factors independently associated with transitioning to a frequent prescription pattern.

**Methods:**

This historical cohort study included patients with active asthma managed in UK primary care and included in the Optimum Patient Care Research Database (OPCRD; opcrd.co.uk). Intermittent OCS prescription patterns were categorized as sporadic, infrequent, moderately frequent, or frequent. Prescription pattern sequences were described for those who had a frequent sequence in their final year of prescribing. We examined associations between OCS prescription pattern and the hazard of transitioning into a frequent intermittent OCS prescription pattern using multivariable Cox regression with a 10-year look-back period.

**Results:**

Of 105,229 patients with intermittent OCS prescriptions, 57.1% (n = 60,083) had a frequent OCS prescription pattern at some point. Irrespective of baseline pattern, most patients transitioned to frequent prescription during the look back. The strongest risk factors were a more frequent prescription pattern at the start of look-back period, a lower percentage peak expiratory flow rate, and higher Global Initiative for Asthma treatment step. Older age, female sex, obesity, and active smoking were also associated with a higher risk of transitioning.

**Conclusion:**

Our findings help identify those most at risk of transitioning to frequent intermittent OCS receipt and encourage earlier intervention with OCS-sparing treatments.

## Introduction

There is a perception that short oral corticosteroid (OCS) courses for acute asthma exacerbations are safe provided they are used infrequently.^1^ Along with failure to optimize inhaled therapy, as well as delay in biologic initiation for patients with severe asthma, this likely contributes to the persistently high prevalence of intermittent OCS prescription for patients with asthma (2.1% to 92.6%).^2^ However, increasing frequency of intermittent OCS prescription and cumulative OCS exposure increase the risk of many OCS-related adverse outcomes (eg, osteoporosis, pneumonia, and type 2 diabetes).^3^

It remains unclear how intermittent OCS prescription patterns (defined by gaps between prescriptions) change over time, whether patients with less frequent intermittent prescription pattern progress to become more frequent, and if there are warning signals that could predict this progression. These gaps in evidence must be bridged before effective clinical strategies can be devised to minimize lifetime exposure to OCS.^4^ Our study aimed to describe the evolution and trajectory of intermittent OCS prescription patterns in patients with asthma and to investigate risk factors independently associated with transitioning to a frequent OCS prescription pattern (indicative of more frequent exacerbations and loss of control).

This was a historical cohort study including patients with active asthma managed in UK primary care and included in the Optimum Patient Care Research Database (OPCRD; opcrd.co.uk). Patients had an OCS prescription with a concurrent asthma event (defined by medication, asthma consultation, and/or diagnosis), a minimum of 1-year pre-OCS baseline data, first OCS prescription on or after January 1, 2000, were ≥18 years old on date of first OCS prescription, and had ≥12 months’ worth of follow-up data. Exclusion criteria have been previously described.^3^ Patient data were extracted between January 1 and December 18, 2019. Patients were followed until the end of their electronic medical record, and those who received therapy with long-term OCS at any point were excluded.

Intermittent OCS prescription patterns were defined as previously described.^5^ Sporadic, infrequent, moderately frequent, and frequent were defined as OCS prescription gaps of ≥365 days, ≥182 to <365 days, ≥90 to <182 days, and <90 days, respectively. We described intermittent OCS prescription pattern sequences in the 5 years before the end of prescribing for those patients who had a frequent sequence in their final year of prescribing. Multivariable Cox regression was used to examine the associations between OCS prescription pattern and hazard of transitioning into a frequent intermittent OCS prescription pattern (first occurrence), adjusting for *a priori*–determined covariates. The look-back period to examine associations extended to 10 years. Patients with a frequent OCS prescription pattern at the start or no active treatment for asthma were excluded from this analysis. Time to first occurrence of frequent intermittent OCS prescription pattern was examined by initial OCS prescription pattern. Stata v14 (StataCorp, College Station, Tex) was used to conduct all statistical analyses.

The study received ethical approval from the Anonymised Data Ethics and Protocol Transparent Committee (ADEPT1120), was performed in compliance with good clinical practice, and was registered with the European Union Electronic Register of Post-authorisation Studies (ENCEPP EUPAS37065) and the Independent Scientific Advisory Committee (ISAC_20_000071).

## Results and discussion

A total of 105,229 patients with an intermittent OCS prescription were included, of whom 57.1% (n = 60,083) had a frequent OCS prescription pattern at some point (see Fig E1 in this article’s Online Repository at www.jaci-global.org). Patients with ever and never frequent OCS receipt had similar demographic and clinical characteristics at baseline, although mean duration of OCS prescriptions was longer and mean cumulative OCS dose was higher in the frequent versus not frequent group (see Table E1 in the Online Repository). Worryingly, 31.4% (n = 18,850/60,083) of patients prescribed frequent intermittent OCS had no prescription for an inhaled corticosteroid. Furthermore, 52.1% (n = 31,272/60,083) had no short-acting β_2_-agonist prescription yet were still prescribed frequent intermittent OCS. A similar pattern of treatment was noted in both the frequent and not frequent groups.

**Fig 1:**
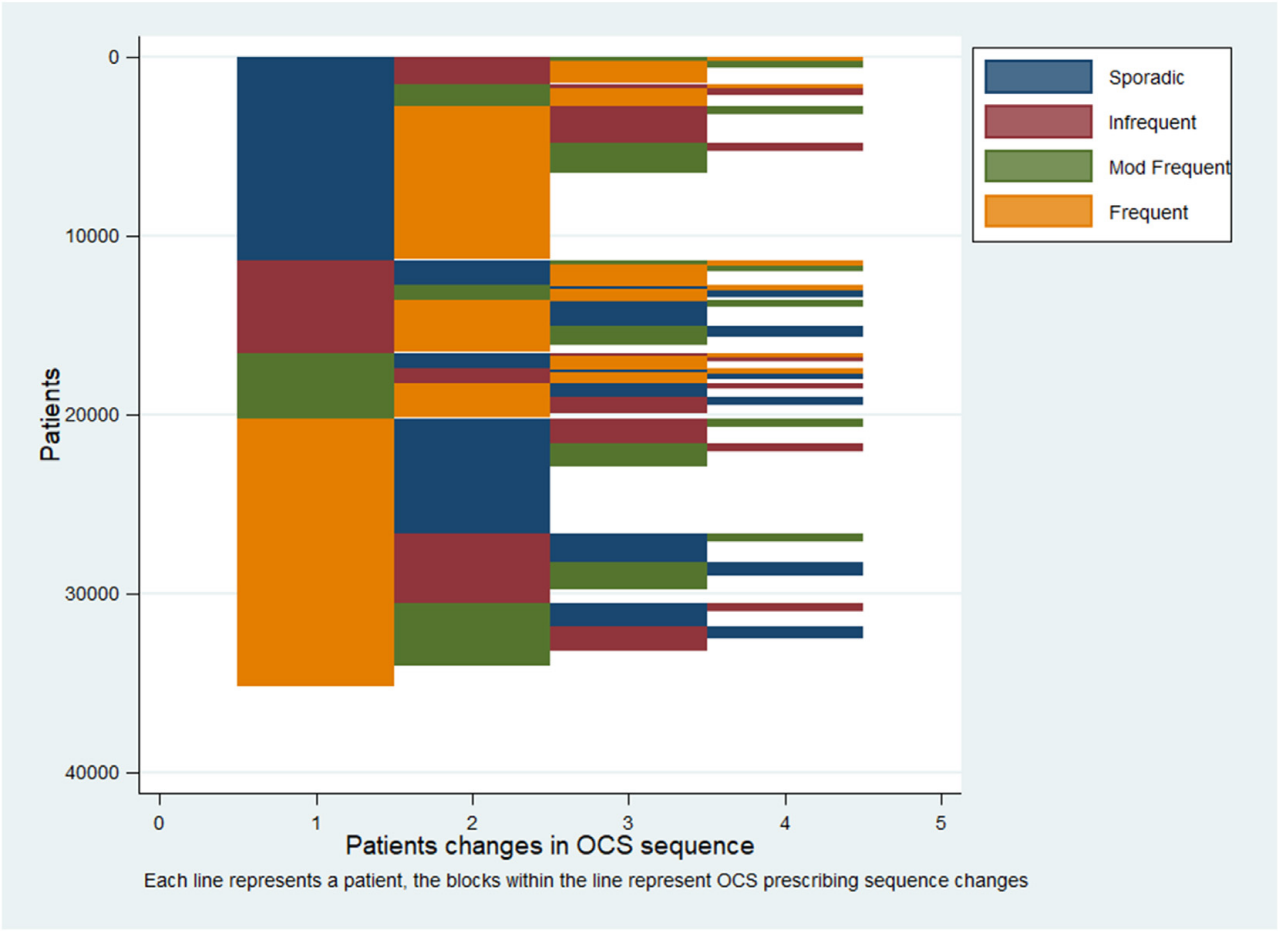
OCS prescribing pattern sequences in final 5 years of prescribing for those with frequent intermittent OCS prescriptions in final year before end of prescribing (n = 35,094). Sporadic, infrequent, moderately frequent, and frequent were defined as OCS prescription gaps of ≥365 days, ≥182 to <365 days, ≥90 to <182 days, and <90 days, respectively. X-axis represents *n*th prescription pattern observed since baseline; y-axis represents, from top, cumulative count of patients. Transition patterns for all patients are summarized, showing that patients transition along an intermittent OCS escalator in numerous ways.

**Fig 2:**
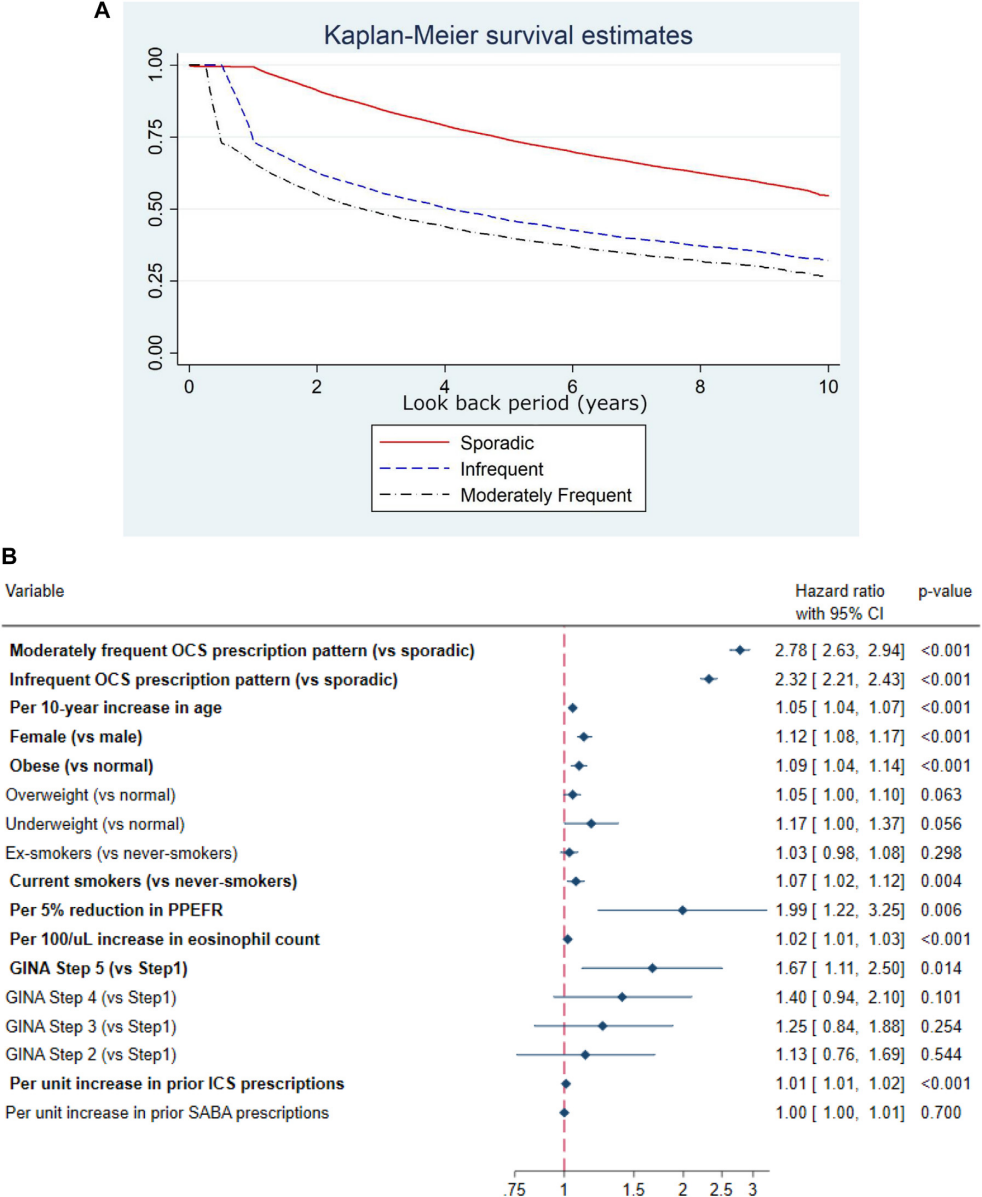
**A,** Cumulative freedom from frequent OCS prescription pattern during a 10-year look-back period, stratified by initial OCS prescription patterns. X-axis represents time since index date in years; y-axis represents cumulative freedom from a frequent OCS prescription pattern. Sporadic, infrequent, and moderately frequent were defined as OCS prescription gaps of ≥365 days, ≥182 to <365 days, and ≥90 to <182 days, respectively. **B,** Associations between potential risk factors and hazards of transitioning into a frequent OCS prescription pattern within a 10-year look-back period. OCS prescription pattern was determined by first 2 prescription after start of look-back period. BMI, smoking status, eosinophil count, and PPEFR were all recorded closest to start of look-back period and within 1 year before start of look-back period. Obese was defined as BMI > 30 kg/m^2^. GINA treatment step as well as total number of ICS and SABA prescriptions were recorded within 1 year before start of look-back period. Per-unit increase of prior SABA prescription relates to 1 prescription. *BMI,* Body mass index; *GINA,* Global Initiative for Asthma; *ICS,* inhaled corticosteroid; *PPEFR,* percentage peak expiratory flow rate; *SABA,* short-acting β_2_-agonist.

The intermittent OCS landscape was complex. The most common OCS prescription patterns were frequent and sporadic for patients who received frequent prescriptions for intermittent OCS in the final year before end of prescribing, and patients moved between intermittent OCS prescription categories in many different ways (Fig 1). Irrespective of baseline OCS prescription pattern, most patients transitioned to frequent intermittent OCS prescription during the 10-year look back. Approximately 75% and 65% of those with a moderately frequent and infrequent prescription pattern at baseline transitioned to frequent OCS prescription pattern, with approximately 25% of these patients doing so in the first year of the look back. This likely reflects poorer asthma control (relative to those with a sporadic OCS prescription pattern) and easier access to repeat OCS prescriptions as per asthma management plans (Fig 2, *A*). In contrast, few patients with a sporadic OCS prescribing pattern at baseline transitioned to a frequent OCS pattern in the first year of the look back, with the proportion gradually increasing to approximately 50% during the 10-year look-back period (Fig 2, *A*). The strongest risk factor for transitioning into a frequent OCS prescription pattern was a more frequent OCS prescription pattern at the start of the 10-year look-back period, followed by a lower percentage peak expiratory flow rate and higher Global Initiative for Asthma treatment step. There was also some evidence that older age, female sex, obesity, and being an active smoker were independently associated with a higher risk of transitioning into a frequent OCS prescription pattern within a 10-year look-back period, although the increased risk was small (Fig 2, *B*). In contrast, there was no evidence of an association with previous receipt of short-acting β_2_-agonist.

Study limitations include the inherent caveats of secondary data, as well as potential unmeasured/residual confounding. OCS prescription was used as a surrogate for OCS receipt, OCS adherence was not measured, and identification of intermittent OCS focused on specificity rather than sensitivity, which may affect generalizability. Study strengths included inclusion of a large patient cohort representative of the white asthma population, investigation of a wide range of *a priori*–identified risk factors for transitioning into a frequent intermittent OCS prescription pattern, and a long look-back period.

To our knowledge, this is the first study to investigate longitudinal changes in intermittent OCS prescription patterns in the United Kingdom. Despite recent therapeutic advances, therapy with OCS remains common across the spectrum of asthma severity.^6^^,^^7^ The situation is aggravated by the continued easy accessibility of OCS worldwide, lack of postexacerbation review, and possible underuse of inhaled corticosteroid therapy and/or poor adherence, as well as the variable nature of inflammation in asthma, suboptimal decision making by physicians, and patient unwillingness to initiate new treatment options.^2^^,^^4^^,^^8^ There is an urgent need to introduce OCS stewardship approaches to encourage investigation of reversible complicating factors in patients receiving OCS, promote consideration of steroid-sparing treatments, and prompt reduction of inappropriate OCS drugs.^4^ Our findings should help physicians identify those most at risk of transitioning to a frequent intermittent OCS prescription pattern (Fig 2, *B*) and encourage closer monitoring and/or review of OCS prescribing practices and earlier intervention with OCS-sparing treatments such as biologics.

## Disclosure statement

Conducted by the Observational and Pragmatic Research Institute (OPRI) Pte Ltd and funded by 10.13039/100004325AstraZeneca Ltd. 10.13039/100004325AstraZeneca funded the study. AstraZeneca and OPRI had a role in study design, data collection, data analysis, data interpretation, and writing the report. The corresponding author had full access to all the data and had final responsibility to submit for publication.

Data sharing: The data set supporting the conclusions of this article was derived from the Optimum Patient Care Research Database (OPCRD; www.opcrd.co.uk). The authors do not have permission to give public access to the study data set; researchers may request access to OPCRD data for their own purposes. Access to OPCRD can be made via the OPCRD website (opcrd.co.uk/our-database/data-requests) or via the enquiries email (info@opcrd.co.uk). The OPCRD has ethical approval from the National Health Service Research Authority to hold and process anonymized research data (Research Ethics Committee reference 15/EM/0150). This study was approved by the Anonymised Data Ethics Protocols and Transparency (ADEPT) committee, which is the independent scientific advisory committee for the OPCRD.

Disclosure of potential conflict of interest: T. N. Tran, J. Chapaneri, B. Emmanuel, D. Gibson, and J. Rowell are employees of and own stock in AstraZeneca. H. Heatley, J. S. K. Chan, and D. Skinner are employees of Observational and Pragmatic Research Institute (OPRI). A. Bourdin has received industry-sponsored grants from AstraZeneca-10.13039/501100004628MedImmune, Boehringer-Ingelheim, 10.13039/100004388Cephalon/Teva, 10.13039/100004330GlaxoSmithKline, Novartis, and Sanofi-Regeneron; and consultancies with AstraZeneca-MedImmune, Boehringer-Ingelheim, GlaxoSmithKline, Novartis, Regeneron-Sanofi, Med-in-Cell, Actelion, Merck, Roche, and Chiesi. D. J. Jackson has received advisory board and speaker fees from AstraZeneca, 10.13039/100004330GlaxoSmithKline, Boehringer Ingelheim, Teva, Napp, 10.13039/100019719Chiesi, and Novartis; and research grant funding from 10.13039/100004325AstraZeneca. A. N. Menzies-Gow is an employee of 10.13039/100004325AstraZeneca and may own stock or stock options in AstraZeneca; has attended advisory boards for 10.13039/100004325AstraZeneca, 10.13039/100004330GlaxoSmithKline, 10.13039/100004336Novartis, 10.13039/100009857Regeneron, 10.13039/100004339Sanofi, and Teva; has received speaker fees from 10.13039/100004325AstraZeneca, 10.13039/100004336Novartis, Teva, and Sanofi; has participated in research with 10.13039/100004325AstraZeneca for which his institution has been remunerated; has attended international conferences with Teva; and has had consultancy agreements with 10.13039/100004325AstraZeneca and 10.13039/100004339Sanofi. R. Murray is a consultant for OPRI (OPRI) which conducted this study in collaboration with Optimum Patient Care and AstraZeneca. D. Price has advisory board membership with 10.13039/100002429Amgen, 10.13039/100004325AstraZeneca, Boehringer Ingelheim, 10.13039/100019719Chiesi, Circassia, 10.13039/100016259Mylan, Mundipharma, 10.13039/100004336Novartis, 10.13039/100009857Regeneron Pharmaceuticals, 10.13039/100013995Sanofi Genzyme, Teva Pharmaceuticals, and Thermo Fisher; consultancy agreements with 10.13039/100002429Amgen, 10.13039/100004325AstraZeneca, Boehringer Ingelheim, 10.13039/100019719Chiesi, 10.13039/100004330GlaxoSmithKline, 10.13039/100016259Mylan, Mundipharma, 10.13039/100004336Novartis, 10.13039/100004319Pfizer, Teva Pharmaceuticals, and Theravance; grants and unrestricted funding for investigator-initiated studies (conducted through OPRI) from 10.13039/100004325AstraZeneca, Boehringer Ingelheim, 10.13039/100019719Chiesi, Circassia, 10.13039/100016259Mylan, Mundipharma, 10.13039/100004336Novartis, 10.13039/100004319Pfizer, 10.13039/100009857Regeneron Pharmaceuticals, 10.13039/100014129Respiratory Effectiveness Group, 10.13039/100013995Sanofi Genzyme, Teva Pharmaceuticals, Theravance, and the UK National Health Service; payment for lectures/speaking engagements from 10.13039/100004325AstraZeneca, Boehringer Ingelheim, 10.13039/100019719Chiesi, Cipla, 10.13039/100004330GlaxoSmithKline, Kyorin, Mylan, Mundipharma, Novartis, Regeneron Pharmaceuticals, Sanofi Genzyme, and Teva Pharmaceuticals; payment for developing educational materials from Mundipharma and Novartis; payment for travel/accommodation/meeting expenses from AstraZeneca, Boehringer Ingelheim, Mundipharma, Mylan, Novartis, and Thermo Fisher; funding for patient enrollment or completion of research from Novartis; stock/stock options from AKL Research and Development Ltd, which produces phytopharmaceuticals; owns 74% of the social enterprise Optimum Patient Care Ltd (Australia and United Kingdom) and 74% of OPRI (Singapore); 5% shareholding in Timestamp, which develops adherence monitoring technology; is peer reviewer for grant committees of the Efficacy and Mechanism Evaluation program and Health Technology Assessment; and was an expert witness for 10.13039/100004330GlaxoSmithKline.

## Supplementary data

Supplementary Fig E1

Supplementary Table E1
