# Supplementary material for: Longitudinal patterns of intermittent oral corticosteroid therapy for asthma in the United Kingdom
Source: J Allergy Clin Immunol Glob. 2024 Feb 2;3(2):100225. doi: 10.1016/j.jacig.2024.100225 (PMC10959664; doi:10.1016/j.jacig.2024.100225)
Supplement: Supplementary Fig E1 [file mmc1.docx]

# Online supplement

**S-Figure 1: Subject disposition**

**
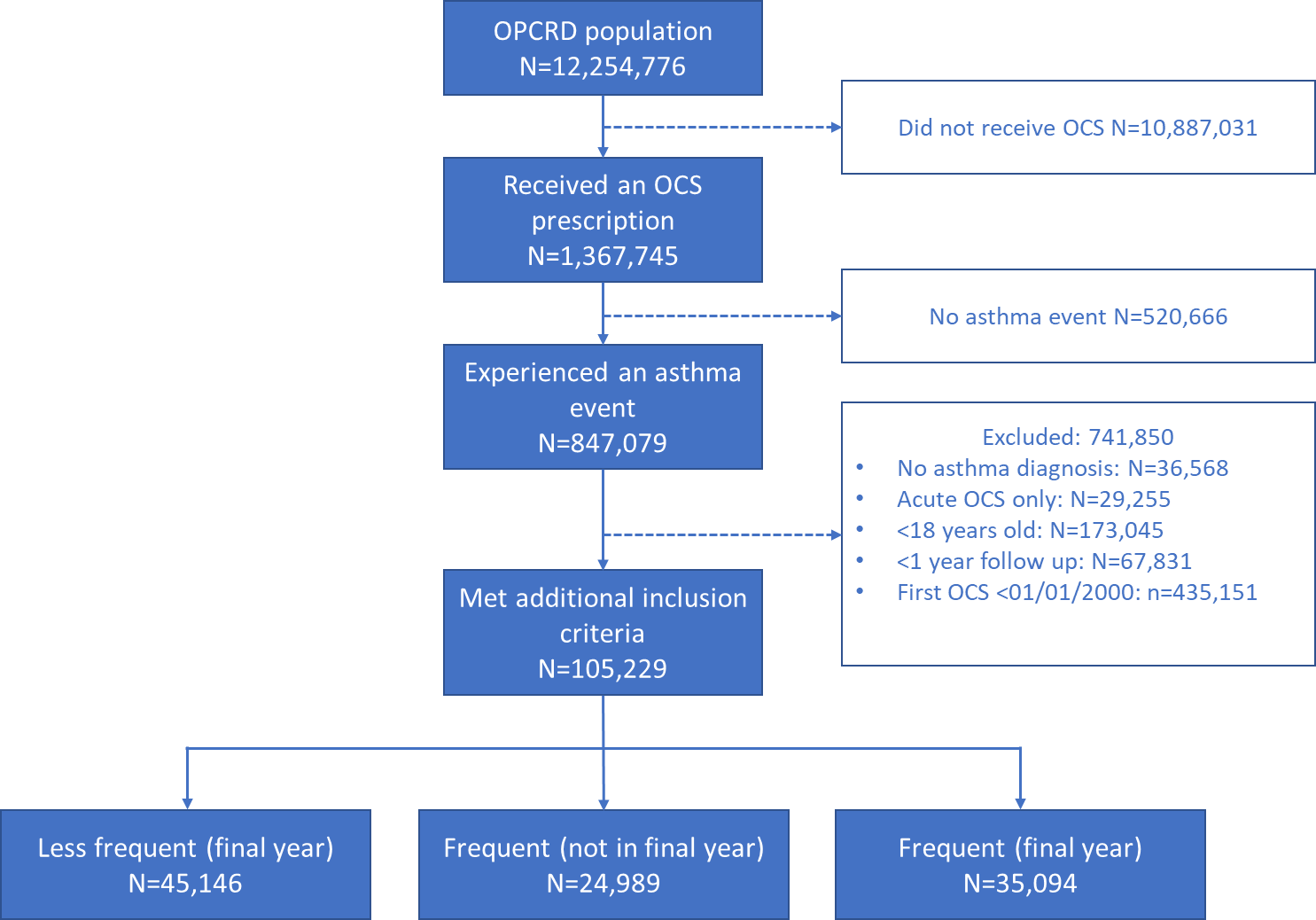
**

**Abbreviations:** OCS: oral corticosteroids; OPCRD: Optimum Patient Care Research Database

Less frequent: OCS prescription gaps of ≥90 days

Frequent: OCS prescription gaps of < 90 days
