# Supplementary material for: Longitudinal patterns of intermittent oral corticosteroid therapy for asthma in the United Kingdom
Source: J Allergy Clin Immunol Glob. 2024 Feb 2;3(2):100225. doi: 10.1016/j.jacig.2024.100225 (PMC10959664; doi:10.1016/j.jacig.2024.100225)
Supplement: Supplementary Table E1 [file mmc2.docx]

# Online supplement

**S-Table 1: Demographic and clinical characteristics at baseline (1 year prior to patients first OCS prescription)**

|  | **Total  (n=105,229)** | **Frequent intermittent OCS at any point during the study**  **N=60,083** | **Not Frequent intermittent OCS during the study N=45,146** |
| --- | --- | --- | --- |
| **Age, years**  Mean (95% CI)  Median (Q1, Q3) | 47.1 (47.0, 47.3)  45.9 (34.2, 59.8) | 48.6 (48.4, 48.7)  47.7 (35.8, 61.4) | 45.2 (45.1, 45.4)  43.7(32.2, 57.4) |
| **Gender**  Male, n (%) | 36,351 (34.5%) | 19,810 (33.0%) | 15,541 (36.6%) |
| **BMI**  Mean (95% CI)  Median (Q1, Q3) | 28.6 (28.5, 28.6)  27.4(23.7, 32) | 28.9 (28.8 28.9)  27.6 (23.9, 32.3) | 28.2 (28.1, 28.3) 27 (23.4, 31.5) |
| **Smoking status**  Never, n (%)  Current, n (%)  Ex-smoker, n (%)  Missing, n (%) | 33,182 (31.5%)  37,299 (35.5%)  26,507 (25.2%) 8,241 (7.8%) | 18,551 (30.9%)  21,209 (35.3%)  15,396 (25.62%)  4,927 (8.2%) | 14,631 (32.4%)  16,090 (35.6%)  11,111 (24.6%)  3,314 (7.3%) |
| **Initial BEC, cells/µL‡**  0 to <150, n (%)  150 to <300, n (%)  300 to < 450, n (%)  >450, n (%)  Missing, n (%) | 26,650 (25.3%)  30,628 (29.1%)  22,595 (21.5%)  15,025 (14.3%)  10,331 (9.8%) | 15,431 (25.7%)  17,452 (29.1%)  13,114 (21.8%)  9,063 (15.1%)  5,023 (8.4%) | 11,219 (24.9%)  13,176 (29.2%)  9,481 (21.0%)  5,962 (13.2%)  5,308 (11.8%) |
| **GINA (2020) treatment step***  No treatment  Step 1, n (%)  Step 2, n (%)  Step 3, n (%)  Step 4, n (%)  Step 5, n (%) | 22,999 (21.9%)  10,637 (10.1%)  34,973 (33.2%)  24,056 (22.9%)  10,446 (9.9%)  2,118 (2.0%) | 13,129 (21.9%)  5,721 (9.5%)  18,398 (30.6%)  14,673 (24.4%)  6,730 (11.2%)  1,432 (2.4%) | 9,870 (21.9%)  4,916 (10.9%)  16,575 (36.7%)  9,383 (20.8%)  3,716 (8.2%)  686 (1.5%) |
| **Number SABA prescriptions**  0, n (%)  1 to <3, n (%)  3 to <12, n (%)  12+, n (%) | 20,684 (19.7%)  47,585 (45.2%)  32,700 (31.1%)  4,260 (4.1%) | 12,349 (20.6%)  26,110 (43.5%)  18,958 (31.6%)  2,666 (4.4%) | 8,335 (18.5%)  21,475 (47.6%)  13,742 (30.4%)  1,594 (3.5%) |
| **Cumulative OCS dose, mg†**  Mean (95% CI)  Median (Q1, Q3) | 1433 (1417, 1450)  780 (450, 1470) | 2092 (2064, 2120)  1250 (770, 2190) | 557 (553, 560)  450 (350, 650) |
| **Duration of OCS prescription, years†**  Mean (95% CI)  Median (Q1, Q3) | 6.41 (6.38, 6.43)  5.4 (2.8, 9.1) | 7.06 (7.02, 7.09)  6.2 (3.3, 10.0) | 5.54 (5.51, 5.58)  4.5 (2.4, 7.8) |

* prior to initiate OCS prescription; † During the study period; ‡ within the last 2 years

**Abbreviations:** BEC: blood eosinophil count; BMI: body mass index; CI: confidence interval; GINA: Global Initiative for Asthma; OCS: oral corticosteroid; SABA: short-acting β_2_-agonist.
